# 

**Published:** 1889-09

**Authors:** 


### TABLE OF CONTENTS.

ORIGINAL AND SELECTED.

Hæmorrhagic malarial fever, By I. T. Young, M D., of La. ... 321
Goitre treated successfully with strophanthus, by S. T. Yount, M. D.; Influence of opium habit on menstruation, by B. M, Wooley, M. D. 328
Monthly summary of med. progress ... 329
The arrest of hemorrhage following excision of the tonsil ... 331
Nitrate of silver in purpura hæmorrhacica; Instantaneous remedy for lumbago ... 332
Management of functional disorders of stomach, by P. R. Cortelyou, M. D. ... 333

ABSTRACTS &c.

Methacetin; Diarrhœa of infants ... 336
Oil of turpentine; Pruritus ani; Caffeine in migraine ... 337
Med. Journals; Mèrcury salts; Elixir of life ... 338
The testicle as a rejuvenator ... 339
Creolin as an antiseptic; Infantile diarrhœa ... 340
Cocaine as a hæmostatic ... 341
Utilizing condemned murderers; Bishop outopsy ... 342
Morphine-vaselin as a local application in cancer; Lobeline in asthma ... 343
Chronic diarrhœa; Injections of lemon juice in epistaxis; Flatulent dyspepsia ... 344
Glycerin in dyspepsia; Arteries of the ox as drainage tubes ... 345
The best antipyretic in febrile tuberculosis; Yellow fever and its prevention; Antidote to morphine poisoning; Chronic sore legs ... 346
A solvent for diphtheric membrane; Typhoid fever from drinking water ... 347
One form of vertigo; Treat. of felons, &c ... 348
Simple treat. for acute coryza; Treat. of hæmoptysis; Antifebrin; Poisoning by glycerine; Removal of warts ... 349

SCIENTIFIC.

Discovery of an Assyrian library three thousand five hundred years old; Cave dwellers found in Mexico ... 350
Photography in prediction of weather; Meteorites; Iron and steel ... 351

NOTES AND FORMULÆ.

Earache; Mouth cavities in carious teeth; Treat. of burns; For constipation ... 352
Treat. of chronic ortorrhœ; Psoriasi capitis; Catarrh; Laxative powder; Hectic fever ... 353

EDITORIAL.

A card; Caution; Case of hydrophobia ... 354
Note from Dr. Gaston; The physician himself; Transactions of the So. Surg. & Gynecological Association ... 356
Bush's fluid food; I Phillips; International Medical congress; Med. Society at Penn; Southern Medical College; Dental department, So. Med. College ... 357
Eleventh annual report of the Presbyterian eye, ear and throat hospital; Convenient methods of medication; Wood's monographs; Treat. of strictures of the urethra by electrolysis; Receipts; Tenth Int. Med. Cong.; American Rhino. Asso.; Miss. Valley Med. Association ... 358
Special notes ... 359, 360

